# Antireflux Surgery in Patients with Moderate Obesity – Fundoplication or Roux-en-Y Gastric Bypass?

**DOI:** 10.1007/s11695-025-07829-1

**Published:** 2025-03-27

**Authors:** Johanna Betzler, Nina Wiegand, Alexandra Kantorez, Alida Finze, Sebastian Schölch, Christoph Reißfelder, Mirko Otto, Susanne Blank

**Affiliations:** 1 Department of Surgery, University Medical Center, Medical Faculty Mannheim, Heidelberg University, Mannheim, Germany; 2 German Cancer Research Center (DKFZ), Heidelberg, Germany; 3 JCCU Translational Surgical Oncology, German Cancer Research Center (DKFZ), Heidelberg, Germany; 4 DKFZ-Hector Cancer Institute at University Medical Center Mannheim, Mannheim, Germany

**Keywords:** Antireflux surgery, Moderate obesity, Fundoplication, RYGB, GERD

## Abstract

**Background:**

Gastroesophageal reflux disease (GERD), often associated with obesity, impairs quality of life and can lead to complications. This study compared Fundoplication and Roux-en-Y Gastric Bypass (RYGB) in patients with WHO (World Health Organization) class I and II obesity and refractory GERD.

**Material and Methods:**

A single-center, retrospective study analyzed 93 patients (55 Fundoplication, 38 RYGB) with BMI < 40 kg/m^2^ who underwent surgery between January 2018 and September 2021. Preoperative characteristics, surgical outcomes, and postoperative results after three months and after one year were analyzed. Quality of life was assessed using Bariatric Quality of Life (BQL) and Quality of Life in Reflux and Dispepsia (QOLRAD) questionnaires. Propensity-score matching (PSM) was performed using the parameters age, BMI and gender.

**Results:**

Patients who underwent RYGB had higher preoperative BMI (35.9 vs. 27.5 kg/m^2^, *p* < 0.0001) and more metabolic comorbidities. Patients who underwent Fundoplication experienced longer anesthesia (192.5 vs. 112 min, *p* < 0.0001), operation times (134 vs. 79 min, *p* < 0.0001), and hospital stays (4 vs. 3 days, *p* = 0.0003). Complication rates in general (*p* = 0.0154, after three months) and dysphagia rates in particular (*p* = 0.0036, after three months and *p* = 0.0147, after one year) were higher in the Fundoplication group. Preoperatively, patients undergoing RYGB reported poorer quality of life in BQL questionnaires (*p* = 0.0008). PSM showed less reflux regression in the Fundoplication group after three months (*p* = 0.0223).

**Conclusion:**

Despite higher preoperative BMI and comorbidities, patients undergoing RYGB had shorter operative times and hospital stays. The results suggest RYGB may be preferable for patients with refractory GERD and class I and II obesity, but further research on long-term outcomes is needed.

**Supplementary Information:**

The online version contains supplementary material available at 10.1007/s11695-025-07829-1.

## Introduction

Gastroesophageal reflux disease (GERD), characterized by the reflux of stomach contents (including mostly acid or bile reflux and less common volume reflux) into the esophagus, significantly impairs quality of life and can lead to complications such as aspiration, esophagitis, Barrett's esophagus, and an increased risk of esophageal adenocarcinoma. Obesity exacerbates the pathophysiological mechanisms of GERD by increasing the intraabdominal pressure, making its management in patients with obesity particularly challenging [1–4].

Surgical interventions, specifically Fundoplication and Roux-en-Y Gastric Bypass (RYGB), have been recognized as effective treatments for patients with GERD refractory to medical management. Fundoplication, which enhances the barrier to reflux by wrapping the upper stomach around the lower esophagus, has been widely used with satisfactory outcomes [5]. On the other hand, RYGB not only aids in significant and sustained weight loss but also alters gastric anatomy and physiology, potentially providing superior control of GERD symptoms in patients with obesity. By creating a small gastric pouch and disconnecting the bile inflow from the stomach, less stomach acids are produced, bile reflux is prevented and the intragastric pressure is reduced due to exclusion of the gastric pylorus [6, 7].

Only few studies have focused on the comparison between Fundoplication and RYGB in patients with WHO (World Health Organization) class III obesity (BMI ≥ 40 kg/m^2^) but it has been shown that RYGB leads to less in-hospital morbidity, more weight loss and better control of concomitant diseases, such as diabetes, hypertension and hypercholesterolemia than Fundoplication [8–10].

In WHO class I and II obesity the choice between Fundoplication and RYGB involves considering several factors, including the severity of GERD symptoms, obesity-related comorbidities, patient preference, and the potential for weight loss and improvement in metabolic syndrome [11].

So far, there is no clear international recommendation concerning the choice of operative technique in patients with refractory GERD and moderate obesity due to the lack of high-quality clinical trials [11–13].

Therefore, this study aims to give profound data on perioperative complications, reflux control, quality of life and reduction of comorbidities after Fundoplication and RYGB in patients with moderate obesity.

## Material and Methods

The trial was conducted as a single-center, retrospective observational study. Ethical approval was obtained from the Ethics Committee II of the Medical Faculty Mannheim at Heidelberg University (reference number 2021–894).

### Inclusion and Exclusion Criteria

Male and female patients aged 20 to 93 years with primary diagnosis of GERD and BMI < 40 kg/m^2^ who underwent RYGB or Fundoplication surgeries between January 2018 and September 2021, at the Surgical Department of University Medical Center Mannheim were included. Patients provided consent for data storage and analysis according to DSGVO (german: Datenschutzgrundverordnung; General Data Protection Regulation) regulations. Patients with WHO class III obesity (BMI ≥ 40 kg/m2) and hiatal hernia without reflux were excluded.

### Data Acquisition

Data were collected from digitized in-hospital medical records, including physician notes, surgery reports, imaging, endoscopy, pH monitoring, manometry, and anesthesia documents.

The database was conducted using REDCap (Research Electroing Data Capture, Eutin, Germany).

### Preoperative Diagnostics

Socio-demographic and clinical information, including BMI, preoperative serum albumin and cholesterol levels, comorbidities, preoperative medications, and diagnostic findings (e.g., endoscopy, manometry, pH monitoring), were recorded. Preoperative questionnaires assessing quality of life and reflux symptoms were administered.

For this the following questionnaires were used: RSI (reflux symptom index) for assessing subjective perception of reflux symptoms [14], BQL (Bariatric Quality of Life) for assessing Quality of Life due to obesity and associated disease [15] and QOLRAD (Quality of Life in Reflux and Dispepsia) for assessing Quality of Life due to reflux symptoms [16, 17].

### Surgical Procedures

Fundoplication surgeries were performed using Toupet (270°), Nissen (360°), or Dor (180°) techniques, while gastric bypass surgeries were Roux-en-Y with gastrojejunostomy with lengths of 60 cm of the biliopancreatic limb and 150 cm of the alimentary limb. During Fundoplication surgeries hiatal repair was usually performed simultaneously. Operations were preferably conducted laparoscopically or using the *DaVinci* (Intuitive Surgical, California, USA) robot. Surgical and anesthesia durations, intraoperative blood loss, and fluid administration were recorded.

### Postoperative Follow-Up

In-hospital postoperative management followed in-hospital standard operating procedures (SOPs) for bariatric surgery or Fundoplication.

Postoperative monitoring, laboratory assessments, and complications were recorded. Follow-up evaluations at three and 12 months postoperatively assessed reflux regression, improvement in comorbidities, weight and BMI changes. Complications were defined by Clavien-Dindo grade and Comprehensive Complication Index (CCI). After at least one year the results of RSI, BQL and QOLRAD questionnaires were recorded again.

### Subgroup Analysis

For valid results, statistical analysis initially was conducted for patients undergoing Fundoplication and all RYGB (including primary RYGB and also conversions of sleeve gastrectomy to RYGB due to refractory reflux disease) and then also for patients undergoing Fundoplication and only primary RYGB.

For better comparison of the patients, a propensity score matching (PSM) was performed, using age, preoperative BMI and gender as matching parameters.

### Statistical Analysis

After extraction of the data from the database they were analyzed by the Institute of Medical Statistics, Medical Faculty Mannheim, Heidelberg University.

Data were analyzed using SAS (Statistical Analysis System) software. Initially, descriptive analysis involved determining absolute and relative frequencies for qualitative variables. Bivariate correlations between variables and surgical procedures (Fundoplication, RYGB, primary RYGB) were examined using Pearson's chi-square test or the Fisher exact test for small sample sizes. Normality of distribution for quantitative features was assessed using the Shapiro–Wilk test. Group comparisons were made using the t-test for normally distributed data and the Mann–Whitney U test for non-normally distributed data. Additionally, the Kruskal–Wallis test was performed for comparisons involving more than two independent samples. The significance level for all tests was set at p < 0.05.

## Results

Between January 2018, and September 2021, 114 patients at the Surgical Department of University Medical Center Mannheim with a BMI < 40 kg/m^2^ underwent operative therapy for refractory reflux disease and/or hiatal hernia, either through RYGB or Fundoplication. Of these 114 patients, six patients received secondary reflux surgery and 15 patients had hiatal hernias without reflux symptoms or measurable gastroesophageal reflux.

**Fig. 1:**
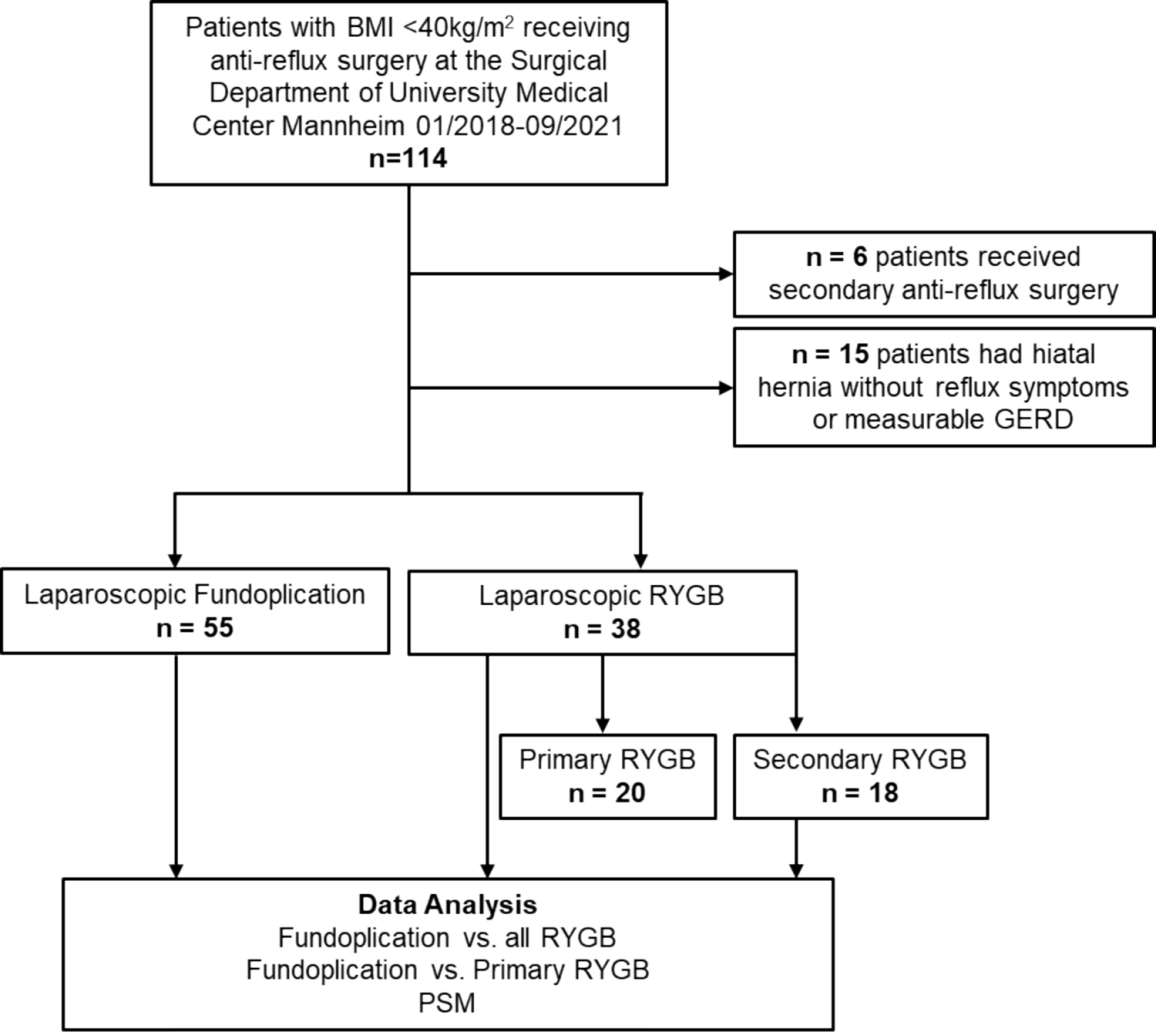
Study flow diagram. BMI = Body Mass Index; RYGB = Roux-en-Y Gastric Bypass; GERD = Gastroesophageal Reflux Disease; PSM = Propensity Score Matching

Of the remaining 93 patients, 55 patients (59.1%) underwent Fundoplication, and 38 patients (40.9%) underwent RYGB, with 20 patients receiving primary RYGB and 18 patients undergoing conversion from a previous sleeve gastrectomy or one-anastomosis gastric bypass (OAGB) to RYGB or revision surgery (n = 3, pouch revision and reconstruction of gastrojejunostomy). The study flow diagram is shown in Fig. 1.

As the results after secondary bariatric surgery are only comparable to a limited extent to those after primary bariatric surgery, a secondary subgroup analysis was performed, including only primary RYGB and patients who underwent Fundoplication.

Regarding Fundoplication, 43 patients received Toupet Fundoplication (78.2%), two patients received Nissen Fundoplication (3.6%), and six patients underwent Dor Hemifundoplication (10.9%).

BMI of the included patients ranged between 17.2 and 39.9 kg/m^2^. Almost two thirds of the patients had either pre-obesity (20.9%) or obesity WHO class I or II (53.5%), 25.6% of the patients were of normal weight. Patients undergoing RYGB had a higher preoperative BMI on average (35.9 kg/m^2^ vs. 27.5 kg/m^2^, *p* < 0.0001) and were heavier on average than patients undergoing Fundoplication (94 kg vs. 75 kg, *p* < 0.0001). Among all patients with obesity, patients undergoing RYGB were more likely to have obesity WHO class II, while patients who underwent Fundoplication were more likely to have obesity class I (*p* = 0.0002).

Preoperatively, metabolic comorbidities such as diabetes mellitus and OSA (obstructive sleep apnea) were significantly more common in patients undergoing RYGB than in patients undergoing Fundoplication (*p* = 0.0132 and *p* = 0.0018, respectively). The majority of the cohort (73.1%) also had a diagnosis of hiatal hernia with significantly more hiatal hernia in the Fundoplication group than in the RYGB group (87.3% vs. 52.6%, *p* = 0.0002). Additionally, 39.8% of the cohort had esophagitis as an additional diagnosis, with no significant difference in frequency between the groups (*p* = 0.7039). However, there was a significant difference in severity, with more patients with higher-grade esophagitis in the Fundoplication group (*p* = 0.0081). Preoperative demographic and clinical characteristics of the cohort are listed in Table 1.

**Table 1:** Demographic and clinical characteristics of the entire cohort

	*Total*	*Surgical Technique*		*p-value*
	n = 93	Fundoplication (n = 55)	RYGB (n = 38)	
*Number of Patients (%)*	93 (100)	55 (59.1)	38 (40.9)	
*Age at Evaluation, Years (mean [SD])*	59 [16.9]	65 [17]	51 [12]	** < 0.0001**
*Gender (%)*				**0.0131**
*Male*	25 (26.9)	20 (36.4)	5 (13.2)	
*Female*	68 (73.1)	35 (63.6)	33 (86.8)	
*Nutritional Status*				
*Preoperative BMI, kg/m*^*2*^* (Med, [IQR])*	31.3 [17.2–39.9]	27.5 [17.2–39.9]	35.9 [23.9–39.2]	** < 0.0001**
Pre-obesity (%)*	18 (20.9)	15 (30.6)	3 (8.1)	**0.0111**
Obesity (%)*	46 (53.5)	13 (26.5)	33 (89.2)	** < 0.0001**
*WHO class I (%)*^*a*^	19 (41.3)	11 (84.6)	8 (24.2)	
*WHO class II (%)*^*a*^	27 (58.7)	2 (15.4)	25 (75.8)	**0.0002**
*Albumin, g/dl (Med, [IQR])*	39.1 [19.4–48]	37.7 [19.4–48]	41.1 [32.9–45.8]	**0.0023**
*Cholesterol, mg/dl (mean, [SD])*	203.1 [48.8]	198.4 [59.6]	207.2 [38.4]	0.6197
*Comorbidities (%)*				
*Cardiovascular*	48 (51.6)	26 (47.3)	22 (57.9)	0.3136
*Pulmonary*	21 (22.6)	13 (23.6)	8 (21.1)	0.7696
*Diabetes mellitus*	12 (12.9)	3 (5.5)	9 (23.7)	**0.0132**
*OSA*	14 (15.1)	3 (5.5)	11 (28.9)	**0.0018**
*Medication (%)*				
*PPI*	56 (60.2)	36 (65.5)	20 (52.6)	0.2143
*Antihypertensives*	40 (43.0)	22 (40.0)	18 (47.4)	0.5272
*Antidiabetics*	8 (8.6)	2 (3.6)	6 (15.8)	0.0595
*Diagnosis (%)*				
*Hiatal Hernia*	68 (73.1)	48 (87.3)	20 (52.6)	**0.0002**
*Barrett’s Esophagus*	2 (2.2)	2 (3.6)	0 (0)	0.5047
*Esophagitis*	37 (39.8)	21 (38.2)	16 (42.1)	0.2669
*LA Stage A*^*b*^	19 (52.8)	11 (55)	8 (50)	
*LA Stage B*^*b*^	8 (22.2)	1 (5)	7 (43.8)	
*LA Stage C*^*b*^	4 (11.1)	3 (15)	1 (6.3)	
*LA Stage D*^*b*^	5 (13.9)	5 (25)	0 (0)	**0.0081**

^*^Calculated based on preoperative documented BMI (n = 64); ^a^ calculated based on “obesity present”; ^b^ calculated based on esophagitis classifiable in Los Angeles (LA) classification (n = 36); *p-value*s < 0.05 are considered significant and marked bold; BMI = Body Mass Index; PPI = Proton Pump Inhibitor; OSA = Obstructive Sleep Apnea; RYGB = Roux-en-Y Gastric Bypass

Preoperative data from quality of life questionnaires (QOLRAD and BQL) and the Reflux Symptom Index (RSI) were available from 48 patients (Table 2). Preoperatively, Patients undergoing RYGB reported poorer quality of life in BQL questionnaire than patients undergoing Fundoplication (*p* = 0.0037). The RSI score was elevated in both groups (≥ 13 points), with an average score of 17.4 in the Fundoplication group and 17.1 in the RYGB group. There was no significant difference between Fundoplication and RYGB in preoperative quality of life assessed by QOLRAD and its individual domains.

**Table 2:** Reflux symptoms and quality of life preoperatively (entire cohort)

	*Total (n* = *48)*	*Fundoplication (n* = *31)*	*RYGB (n* = *17)*	*p-value*
*RSI-Score (Mean [SD])*	17.2 [10.1]	17.4 [9.7]	17.1 [11.2]	0.8236
*BQL Mean Value (Mean [SD])*	3.1 [0.9]	3.4 [0.6]	2.5 [1.0]	0.0008*
*QOLRAD Mean Value (Median [IQR])*	3.8 [1.9–7]	3.4 [1.9–7]	4.1 [1.9–6.7]	0.4544*
*QOLRAD Domains, Sum Scores (Median [ICR])*				
*Domain 1: Emotional Distress*	21.5 [8–42]	21 [12–42]	25 [8–42]	0.4413*
*Domain 2: Sleep Problems*	18 [5–35]	17.5 [6–35]	20 [5–35]	0.6631*
*Domain 3: Vitality*	10 [4–21]	9.5 [4–21]	11 [4–21]	0.4391*
*Domain 4: Eating/Drinking Problems*	18 [8–42]	18 [8–42]	19.5 [12–42]	0.3775*
*Domain 5: Psychological/Emotional Functionality*	21.5 [10–35]	19 [10–30]	24 [10–35]	0.4182*

*p-value*s < 0.05 are considered significant and marked bold. BQL = Bariatric Quality of Life; QOLRAD = Quality of Life in Reflux and Dyspepsia; RSI = Reflux Symptom Index; RYGB = Roux-en-Y Gastric Bypass

### Operation Data and in-Hospital Stay

Table 3 provides an overview of intraoperative and postoperative in-hospital data. 94% of the cohort underwent laparoscopic surgery while 6% of patients underwent surgery with the *DaVinci* robot.

**Table 3:** Intra- and postoperative data of the entire cohort

	*Total (n* = *93)*	*Fundoplication (n* = *55)*	*RYGB (n* = *38)*	*p-value*
*Anesthesia Duration (Minutes) (Median [IQR])*	169 [59–366]	192,5 [103–366]	112 [59–243]	** < 0.0001**
*Surgery Duration (Minutes) (Median [IQR])*	110 [46–297]	134 [58–297]	79 [46–195]	** < 0.0001**
*Blood Loss (ml) (Median [IQR])*	50 [0–200]	50 [0–200]	50 [50–150]	0.8869
*Hospital Stay (Days) (Median [IQR])*	4 [2–50]	4 [2–50]	3 [2–14]	**0.0003**
*Surgical Approach (%)*				1.0000
*- Laparoscopic*	87 (94.5)	51 (92.7)	36 (94.7)	
*- DaVinci*	6 (6.5)	4 (7.3)	2 (5.3)	
*Complications (%)*				
*- Intraoperative*	3 (3.2)	2 (3.6)	1 (2.6)	1.0000
*- Postoperative in Hospital*	21 (22.6)	11 (20)	10 (26.3)	0.4739
*- Surgical*	13 (14)	5 (9.1)	8 (21.1)	0.1020
*- Medical*	8 (8.6)	7 (12.7)	1 (2.6)	0.1352
*Reoperation (%)*	7 (7.5)	2 (3.6)	5 (13.2)	0.1175
*Clavien-Dindo Classification (%)*				0.4633
*- 0*	72 (77.4)	44 (80)	28 (73.7)	
*- 1*	9 (9.7)	6 (10.9)	3 (7.9)	
*- 2*	2 (2.1)	1 (1.8)	1 (2.6)	
*- 3a* + *3b*	9 (9.7)	3 (5.5)	6 (15.8)	
*- 4a* + *4b*	1 (1.1)	1 (1.8)	0 (0)	
*CCI (Median [IQR])*	0 [0–56.1]	0 [0–56.1]	0 [0–39.7]	0.4130

*p-value*s < 0.05 are considered significant and marked bold. CCI = Comprehensive Complication Index; RYGB = Roux-en-Y Gastric Bypass

Three patients experienced intraoperative complications, two of whom underwent Fundoplication. During the two Fundoplication surgeries with intraoperative complications, there occurred one pleural and one esophageal injury. The only intraoperative complication in the RYGB group was a small bowel injury.

During the hospital stay, seven revisional surgeries had to be performed (7.5% in total). In the Fundoplication group two revisional surgeries were performed due to a recurrent hiatal hernia and due to a cardia stenosis. In the RYGB group, five revisional surgeries were performed: due to small intestine perforation (n = 2), anastomotic leak of the gastrojejunostomy (n = 1), anastomotic stenosis (n = 1) and postoperative bleeding with intraabdominal hematoma (n = 1). In-hospital mortality was 0% (n = 0).

Patients undergoing Fundoplication had significantly longer anesthesia (*p* < 0.0001) and operation times (*p* < 0.0001) as well as longer hospital stay (*p* = 0.0003) in comparison to patients undergoing RYGB.

### Follow-Up Course of the Entire Cohort

Tables 4 and 5 provide an overview of follow-up courses at three months and one year postoperatively in the entire cohort (including primary and secondary RYGB). Percentages refer to patients who underwent follow-up, unless otherwise stated. Of the patients undergoing Fundoplication, 33 patients (60.0%) took part in the three-month follow-up, after one year 46 of the patients (83.6%) undergoing Fundoplication attended follow-up. Of the 38 patients undergoing RYGB, 37 (97.4%) participated in follow-up.

**Table 4:** Postoperative outcomes of the entire cohort at 3-month follow-up

	*Total (n* = *70)*	*Fundoplication (n* = *33)*	*RYGB (n* = *37)*	*p-value*
*Reflux Regression (%)*	55 (78.6)	23 (69.7)	32 (86.5)	0.0875
*Nutritional Status*				
*BMI, kg/m*^*2*^* (Mean [SD])*	29.1 [4.3]	24.7 [4.1]	30.3 [3.5]	**0.0004**
*BMI, kg/m*^*2*^* Difference (Median [IQR])*	−4 [−12–2]	−1 [−2–2]	−6 [−12-(−1)]	**0.0003**
*Weight, kg (Mean [SD])*	79.4 [13.6]	70.1 [10.4]	81.9 [13.4]	**0.0339**
*Weight Difference, kg (Mean [SD])*	−12.4 [8.9]	−2.3 [4.2]	−14.8 [8]	**0.0003**
*%TWL (Median [IQR])*	- 11.4 [−19.2-(−6.6)]	−7.1 [−7.7-(−2.8)]	−16.0 [22.1-(−8.0)]	**0.0038**
*Complications (%)*	20 (28.6)	14 (42.4)	6 (16.2)	**0.0154**
*Dysphagia (%)*	7 (10)	7 (21.2)	0 (0)	**0.0036**
*Need for Reoperation (%)*	6 (8.6)	4 (12.1)	2 (5.4)	0.4107
*Clavien-Dindo Classification (%)*				**0.0337**
*Grade 1*	11 (55)	10 (71.4)	1 (16.7)	
*Grade 2*	2 (10)	0 (0)	2 (33.3)	
*Grade 3a* + *3b*	7 (35)	4 (28.6)	3 (50)	

*p-value*s < 0.05 are considered significant and marked bold. BMI = Body Mass Index; RYGB = Roux-en-Y Gastric Bypass; %TWL = Percentage Total Weight Loss

**Table 5:** Postoperative outcomes of the entire cohort at 1-year follow-up

	*Total (n* = *81)*	*Fundoplication (n* = *46)*	*RYGB (n* = *37)*	*p-value*
*Reflux Regression (%)*	60 (74.1)	30 (68.2)	30 (81.1)	0.1195
*Nutritional Status*				
*BMI kg/m*^*2*^* (Mean [SD])*	27.1 [5.2]	26.7 [5.4]	27.5 [5]	0,5126
*BMI kg/m*^*2*^* Difference (Median [IQR])*	−2 [−18–7]	0 [−18–7]	−9 [−17–3]	** < 0.0001**
*Weight kg (Mean [SD])*	75.6 [18.5]	74.5 [18.9]	76.9 [18.1]	0.8325
*Weight kg Difference (Median [IQR])*	−3.5 [−43–19]	−1 [−40–19]	−18 [−43–9]	**0.0002**
*%TWL (Median [IQR])*	−6.7 [−25.3–0]	−1.2 [−5.8–1.9]	−24.3 [−34.8-(−9.1)]	** < 0.0001**
*Complications (%)*	22 (27.2)	9 (20.5)	13 (35.1)	0.1187
*Dysphagia (%)*	7 (8.6)	7 (15.9)	0 (0)	**0.0147**
*Need for Reoperation (%)*	10 (12.3)	3 (6.8)	7 (18.9)	0.1042
*Clavien-Dindo Classification* (*%)*				0.8227
*Grade 1*	10 (45.45)	5 (55.6)	5 (38.4)	
*Grade 2*	2 (9.1)	1 (11.1)	1 (7.7)	
*Grade 3a* + *3b*	10 (45.45)	3 (33.3)	7 (53.9)	

*p-value*s < 0.05 are considered significant and marked bold. BMI = Body Mass Index; RYGB = Roux-en-Y Gastric Bypass; %TWL = Percentage Total Weight Loss

After three months, patients who underwent Fundoplication experienced more complications than patients who underwent RYGB (*p* = 0.0154). In contrast to this finding, patients undergoing RYGB had significantly higher Clavien-Dindo grades at the three-month follow-up than patients undergoing Fundoplication (*p* = 0.0337). These significant differences of complications in general and Clavien-Dindo grading diminished at the one-year follow-up (*p* = 0.1187 and *p* = 0.8827).

The most common complications in the Fundoplication group at the three-month follow-up were severe epigastric pain (n = 4), dysphagia (n = 6), and severe reflux (n = 4). In the RYGB group, one patient reported severe epigastric pain and two patients reported diarrhea and nausea. One patient suffered from an early anastomotic ulcer which was treated with proton pump inhibitors, one patient had an anastomotic stenosis with subsequent endoscopic balloon dilatation and one patient suffered from an anastomotic leak of the gastrojejunostomy with subsequent laparoscopic overstitching.

Significantly more patients who underwent Fundoplication reported dysphagia at the three-month follow-up compared to patients who underwent RYGB (*p* = 0.0036). This difference persisted after one year (*p* = 0.0147).

In the entire cohort, reflux symptoms regressed in 74.1% of patients after one year. Patients undergoing RYGB showed a tendency towards more regression of symptoms compared to the Fundoplication group after one year (*p* = 0.0875).

The significantly worse results of the BQL questionnaire in the RYGB group diminished in the postoperative follow-up (*p* = 0.3016) and patients undergoing RYGB showed significantly stronger improvement in BQL (*p* = 0.0005). The RYGB and Fundoplication groups showed no significant differences in RSI and QOLRAD results after three months and one year.

As expected, patients who underwent RYGB lost more body weight on average at three months and one year compared to patients who underwent Fundoplication (*p* = 0.0003 and *p* = 0.0002). At one year follow-up, there was no significant difference in BMI between Fundoplication and RYGB (*p* = 0.5126).

### Follow-Up Course Of Fundoplication and Primary RYGB

As secondary bariatric surgery and primary bariatric surgery can only be compared to a limited extent, a secondary analysis of follow-up data was performed. In this analysis of three-month and one-year follow-up only patients with primary RYGB (n = 19) and patients with Fundoplication (n = 33) were included.

As already seen in the entire cohort, patients who received primary RYGB lost significantly more weight (*p* < 0.0001 and *p* < 0.0001) and BMI (*p* = 0.0002 and *p* < 0.0001) than patients undergoing Fundoplication after three months and one year.

Patients who underwent Fundoplication had significantly higher complication rates in general and more dysphagia at three-months follow-up than patients who underwent RYGB (*p* = 0.0487 and *p* = 0.039). This effect diminished after one year (*p* = 0.1008 and *p* = 0.0882). Reflux regression, revisional surgery rates and Clavien-Dindo grading did not differ between the two groups.

### Propensity Score Matching

Patients from both groups were paired based on propensity score matching (PSM) for age, preoperative BMI, and gender. PSM resulted in 13 pairs. Table 7 (supplemental material) provides an overview of patient preoperative data after PSM. There were no significant differences between the two groups in preoperative data or questionnaire scores. An overview of data after PSM in follow-up examinations at three months and one year can be found in Table 6.

**Table 6:** Follow-up data after propensity score matching

	*Total (n* = *26)*	*Fundoplication (n* = *13)*	*RYGB (n* = *13)*	*p-value*
*Reflux Regression (%)*				
*After 3 Months*	13 (76.5)	1 (25)	12 (92.3)	**0.0223**
*After 1 Year*	19 (82.6)	8 (72.7)	11 (91.7)	0.3168
*BMI Difference kg/m*^*2*^* (Med [Range])*				
*After 3 Months*	−6 [−10–2]	2 [2–2]	−6 [−10-(−1)]	0.1116
*After 1 Year*	−1 [−18–7]	0 [−18–7]	−5.5 [−14–3]	0.1444
*Postoperative RSI Score (Med [Range])*	10 [0–36]	14 [0–36]	3 [0–15]	0.0616
*RSI Difference (Med [Range])*	−8 [−25–20]	−4 [−22–20]	−15 [−25–3]	0.0817
*Postoperative BQL Average (Med [Range])*	4.4 [2.2–4.8]	3.6 [2.2–4.5]	4.6 [2.8–4.8]	**0.0183**
*Postoperative QOLRAD Average (Med [Range])*	5.5 [3–7]	4.9 [3–7]	6.1 [4.1–7]	0.2366
*Postoperative QOLRAD Sum Scores (Med [Range])*				
*Domain 1: Emotional Distress*	37.5 [16–42]	31 [16–42]	38 [23–42]	0.4196
*Domain 2: Sleep Problems*	31 [13–35]	23 [13–35]	35 [28–35]	**0.0309**
*Domain 3: Vitality*	16 [8–21]	13 [8–21]	19 [10–21]	0.2176
*Domain 4: Eating/Drinking Problems*	28.5 [12–42]	27 [14–42]	35 [12–42]	0.5548
*Domain 5: Psychological/Emotional Functionality*	33 [14–35]	32 [14–35]	34 [24–35]	0.5438
*Complications after 3 Months (%)*^*a*^	5 (29.4)	2 (50)	3 (23.1)	0.5378
*Clavien-Dindo Classification after 3 Months (%)*				1.0000*
*Grade 1*	3 (60)	2 (100)	1 (33.3)	
*Grade 2*	1 (20)	0 (0)	1 (33.3)	
*Grade 3a* + *3b*	1 (20)	0 (0)	1 (33.3)	
*Complications after 1 Year (%)*^*a*^	4 (17.4)	2 (18.2)	2 (16.7)	1.0000
*Clavien-Dindo Classification after 1 Year (%)*				1.0000
*Grade 1*	3 (75)	1 (50)	2 (100)	
*Grade 2*	1 (25)	1 (50)	0 (0)	

^a^ Refers to patients who attended the respective follow-up; *p-value*s < 0.05 are considered significant and marked bold. BMI = Body Mass Index; RSI = Reflux Symptom Index; RYGB = Roux-en-Y Gastric Bypass; BQL = Bariatric Quality of Life; QOLRAD = Quality of Life in Reflux and Dyspepsia

After three months, reflux symptoms in the Fundoplication group regressed significantly less compared to the RYGB group (25% vs. 92.3%, *p* = 0.0223). After one year, the difference was no longer significant (72.7% vs. 91.7%, *p* = 0.3168). Nevertheless, there was a trend towards higher RSI scores at one-year follow-up in the Fundoplication group (14 points vs. 3 points, *p* = 0.0616).

Patients undergoing RYGB had significantly higher quality of life in the BQL questionnaire postoperatively (*p* = 0.0183). Another significant difference was found in postoperative QOLRAD Domain 2, which represents sleep problems. Within this domain, the total score of the Fundoplication group was significantly lower than in the RYGB group (*p* = 0.0309).

## Discussion

The presented study provides critical insights into the management of gastroesophageal reflux disease (GERD) in patients with moderate obesity (WHO class I and II), comparing the outcomes of laparoscopic Fundoplication and Roux-en-Y gastric bypass (RYGB). The choice between these surgical options has been a topic of debate, particularly given the lack of explicit recommendations in international guidelines [11].

The findings from this retrospective analysis underscore the differential impacts of Fundoplication and RYGB on GERD symptoms, weight loss, and quality of life. Consistent with previous literature, RYGB has demonstrated superior efficacy in weight reduction and the control of obesity-related comorbidities, including GERD, in patients with morbid obesity [8–10]. Our study extends these observations to patients with moderate obesity, revealing significant improvements in weight loss and GERD symptom regression following RYGB compared to Fundoplication.

Obesity often goes along with reduction of quality of life due to lack of self-confidence, physical restrictions and emotional distress [18]. Obesity is a crucial factor in the assessment of quality of life and should be taken into account whilst choosing the operative technique for patients with obesity and GERD.

Patients that received RYGB were heavier than patients who received Fundoplication and showed impaired quality of life in the BQL questionnaire before surgery. Fortunately, the BQL difference diminished after surgery. The obesity-related quality of life increased significantly more in the RYGB group than in the Fundoplication group. In regard to reflux symptoms and reflux-related quality of life, the subjective assessment showed no difference between the two groups.

In the analyzed study cohort, there was no significant difference in intraoperative complications but the length of surgery and anesthesia as well as the length of hospital-stay were significantly higher in the Fundoplication group. Especially for patients with several comorbidities and therefore increased risk of perioperative complications, this could be a reason for choosing RYGB over Fundoplication.

The analysis of the follow-up course was done twice: once for Fundoplication and all RYGB and once for Fundoplication and only primary RYGB. The reason for the latter analysis was that the RYGB group primarily contained also patients who received a transformation of sleeve gastrectomy to RYGB due to reflux symptoms and therefore were more likely to have a complicated postoperative course than patients who received their first surgery for GERD and/or obesity.

As already mentioned, we could show a greater weight and BMI reduction after RYGB than after Fundoplication, which coincides with previous studies. This significant effect could be seen in the entire cohort after three months and one year and also in the cohort with only primary RYGB included. Weight reduction in patients with pre-obesity or obesity WHO class I and II is a very important part of the effects of RYGB. Although in Germany there is no consensus on bariatric surgery in patients with GERD and obesity WHO class I and II without severe obesity-related comorbidities, in other countries bariatric surgery is already recommended for patients with lower BMI [19]. Obesity WHO class I is already associated with increased risk of nonalcoholic fatty liver disease, obstructive sleep apnea (OSA), polycystic ovary syndrome and bone and joint disease [20–23]. Also, it has been shown that obesity is an important risk factor for certain cancers [24]. Therefore, the aspect of weight loss due to RYGB has to be taken into account during the choice of operative technique for patients with moderate obesity and GERD, especially if they already have obesity-related comorbidities.

In the entire cohort a higher complication rate after three months in the Fundoplication group could be shown that was not significant anymore one year after surgery. Interestingly, we saw significantly more dysphagia in patients after Fundoplication than after RYGB. This persisted after one year. The higher rate of dysphagia might be due to technical aspects of the Fundoplication technique, as the fundus is wrapped around the distal esophagus and fixed with sutures. Although most Fundoplications were done by the technique by Toupet where the fundus is not wrapped completely around the distal esophagus to prevent tightness at the gastroesophageal junction, dysphagia still seems to be a major problem after Fundoplication [25–27].

Although the complication rate was significantly higher in the Fundoplication group after three months, the distribution of Clavien-Dindo grades was contradictory. In the RYGB group there were significantly higher Clavien-Dindo grades than in the Fundoplication group. This might be due to different diagnostic and therapeutic pathways after the two different operative techniques. Patients after Fundoplication often suffer from dysphagia and pain during food intake, which can be treated with analgesics but not often requires invasive diagnostic or therapeutic interventions [25, 28]. After RYGB patients might suffer from abdominal pain or vomiting which often leads to early invasive diagnostic (e.g. esophago-gastro-duodenoscopy) or therapeutic interventions (e.g. diagnostic laparoscopy if previous diagnostics showed signs of internal hernia) [29, 30].

With the propensity score matching, there was better reflux control after three months after RYGB than after Fundoplication. This effect diminished after one year. The worse results of Domain 2 of the QOLRAD questionnaire (sleep problems) in the Fundoplication group might be due to the above-mentioned higher dysphagia rate of this group.

The presented study has some limitations. With 93 patients in total and only 20 patients who received primary RYGB, the patient cohort is relatively small, and the data validity is limited. A propensity-score matching leads to higher quality of results but with only 13 matching pairs the informative value of these results is also limited, and not significant results might be due to the limited case numbers. The retrospective character of the study is another limitation.

In conclusion, this study contributes valuable insights into the ongoing debate regarding the optimal surgical strategy for managing refractory GERD in the context of moderate obesity. It highlights the necessity of considering a broader range of outcomes, including weight loss, GERD symptom control, complication rates, and quality of life improvements, in making informed clinical decisions.

Due to the results of this study it can be assumed that a randomized clinical trial comparing Fundoplication and RYGB for GERD in patients with moderate obesity might show higher complication grades after RYGB than after Fundoplication because of the higher risks of postoperative bleeding, anastomotic ulcers and internal hernia. Despite this possibly higher risk of postoperative complications with the necessity of reintervention after RYGB, these complications are mostly better manageable than persistent dysphagia or reflux symptoms after Fundoplication. Our data suggest an even better reflux control in patients with obesity after RYGB than after Fundoplication, although the data quality is not sufficient to confirm this hypothesis. Nevertheless, the highly positive effects of weight loss and reduction of comorbidities after this bariatric procedure must be acknowledged.

Further prospective, randomized-controlled studies comparing Fundoplication and RYGB in patients with moderate obesity should be conducted to obtain valid results concerning reflux control, weight loss, reduction of comorbidities and improvement of quality of life. With these studies a consistent national recommendation for the choice of operative technique for patients with refractory GERD and class I and II obesity could be developed.

## Supplementary Information

Below is the link to the electronic supplementary material.

Supplementary file1 (DOCX 16 KB)

## Data Availability

Data is provided within the manuscript or supplementary material.
