# Supplementary material for: Antireflux Surgery in Patients with Moderate Obesity – Fundoplication or Roux-en-Y Gastric Bypass?
Source: Obes Surg. 2025 Mar 27;35(5):1750–60. doi: 10.1007/s11695-025-07829-1 (PMC12065731; doi:10.1007/s11695-025-07829-1)
Supplement: Supplementary file 1 — Supplementary file1 (DOCX 16 KB) [file 11695_2025_7829_MOESM1_ESM.docx]

**Supplementary Material**

**Table 7:** Preoperative data after PSM

|  | **Total** | **Fundoplication (n=13)** | **RYGB (n=13)** | **p-value** |
| --- | --- | --- | --- | --- |
| Age Years (Mean [SD]) | 59 [12.3] | 59.5 [13.9] | 58.5 [10.8] | 0.8400 |
| Gender (%) |  |  |  | 1,0000 |
| - Male | 4 (15.4) | 2 (15.4) | 2 (15.4) |  |
| - Female | 22 (84.6) | 11 (84.6) | 11 (84.6) |  |
| Preoperative BMI kg/m^2^ (Mean [SD]) | 32.6 [3.8] | 32.5 [3.7] | 32.7 [4.1] | 0.9286 |
| Preoperative RSI Score (Med [Range]) | 16 [2-40] | 16 [4-34] | 16 [2-40] | 0.9277 |
| Preoperative BQL Average (Med [Range]) | 3.1 [2.1-4.5] | 3.4 [2.2-4.2] | 2.9 [2.1-4.5] | 0.4109 |
| Preoperative QOLRAD Average (Med [Range]) | 3.4 [1.9-6.7] | 2.9 [1.9-6.2] | 4.4 [2.6-6.7] | 0.1739 |
| Preoperative QOLRAD Sum Scores (Med [Range]) |  |  |  |  |
| - Domain 1: Emotional Distress | 20 [12-40] | 19 [12-40] | 25 [16-42] | 0.1866 |
| - Domain 2: Sleep Problems | 17.5 [5-35] | 15 [7-34] | 22 [5-35] | 0.2956 |
| - Domain 3: Vitality | 10 [4-21] | 9 [4-18] | 13 [7-21] | 0.1727 |
| - Domain 4: Eating/Drinking Problems | 18 [8-42] | 16 [8-35] | 21 [16-42] | 0.0913 |
| - Domain 5: Psychological/Emotional Functionality | 20.5 [10-35] | 17 [10-34] | 27 [18-35] | 0.1029 |

p-values <0.05 are considered significant and marked bold. RSI = Reflux Symptom Index; BQL = Bariatric Quality of Life; QOLRAD = Quality of Life in Reflux and Dyspepsia.
